# The effect of melatonin on irritable bowel syndrome patients with and without sleep disorders: a randomized double-blinded placebo-controlled trial study

**DOI:** 10.1186/s12876-023-02760-0

**Published:** 2023-04-25

**Authors:** Masood Faghih Dinevari, Farzaneh Jafarzadeh, Amirreza Jabbaripour Sarmadian, Samaneh Abbasian, Zeinab Nikniaz, Ali Riazi

**Affiliations:** Liver and Gastrointestinal Diseases Research Center, Tabriz University of Medical Sciences, Tabriz, Iran

**Keywords:** Irritable Bowel Syndrome, Melatonin, Sleep disorders

## Abstract

**Background:**

Irritable bowel syndrome (IBS) is one of the world's most common gastrointestinal (GI) disorders, and current treatments do not meet patients' demands. This study aimed to investigate melatonin's therapeutic effects on IBS score, GI symptoms, quality of life, and sleep parameters in both groups of IBS patients with and without sleep disorders.

**Methods:**

In this randomized double-blinded placebo-controlled trial study, 136 patients with a diagnosis of IBS based on ROME IV criteria were enrolled and then divided into two groups respecting having sleep disorders or not. Patients of each group were randomized in a 1:1 ratio to receive melatonin 6 mg daily (3 mg fasting and 3 mg at bedtime) for 2 months (8 weeks). Blocked randomization was used in this process. All patients were evaluated both at the beginning and the end of the trial regarding IBS score, GI symptoms, quality of life, and sleep parameters through valid questionnaires.

**Results:**

In both groups of patients with and without sleep disorders, a significant improvement was observed in IBS score and GI symptoms, including the severity and the frequency of abdominal pain, the severity of abdominal bloating, satisfaction with bowel habits, disease's impact on patient's life, and stool consistency; however, there was no significant improvement in the frequency of defecations per week. In patients with sleep disorders, significant improvement in sleep parameters, including subjective sleep quality, sleep latency, sleep duration, sleep efficiency, and daytime dysfunction, was observed, while in patients without sleep disorders, there was no significant improvement in sleep parameters. In addition, quality-of-life improvement was observed in a significant number of melatonin recipients compared to placebo in both groups of patients.

**Conclusion:**

Melatonin can be considered an effective treatment for improving IBS score, GI symptoms, and quality of life in IBS patients with and without sleep disorders. It is also effective to improve sleep parameters in IBS patients with sleep disorders.

**Trial registration:**

This study has been registered to the Iranian Registry of Clinical Trials (IRCT) with the approval number IRCT20220104053626N2 on the date of 13/02/2022.

## Introduction

Irritable bowel syndrome (IBS) is one of the world's most common gastrointestinal (GI) disorders. The estimated prevalence of this disease is 11.2% globally and varies based on the country, age, gender, and diagnostic criteria. It significantly affects patients' quality of life and social activities, and its main symptoms are abdominal pain, changes in stool consistency, and defecation frequency [1–3].

The etiology and pathophysiology of IBS are not fully understood, and it is considered a multifactorial disorder [1, 2]. Several studies have been conducted on the role of melatonin in the pathogenesis of IBS and its therapeutic effects [4]. Clinical trials conducted on the therapeutic effects of melatonin showed that melatonin could be a promising treatment for IBS patients in improving GI symptoms and quality of life [5–8]; however, there were no significant effects on the sleep parameters of these patients [5, 6]. It should be noted that these trials studied the sleep parameters in IBS patients with sleep disorders, and no study has studied the impacts of melatonin on these parameters in patients without sleep disorders.

Previous trials have limitations such as small numbers of participants, gender limitations, and not using the latest IBS diagnostic criteria. Therefore, according to the limitations of previous studies and the high prevalence of this disease, this trial aimed to investigate the therapeutic effects of melatonin on IBS score, GI symptoms, quality of life, and sleep parameters in IBS patients both with and without sleep disorders.

## Methods

### Study design

**Fig. 1:**
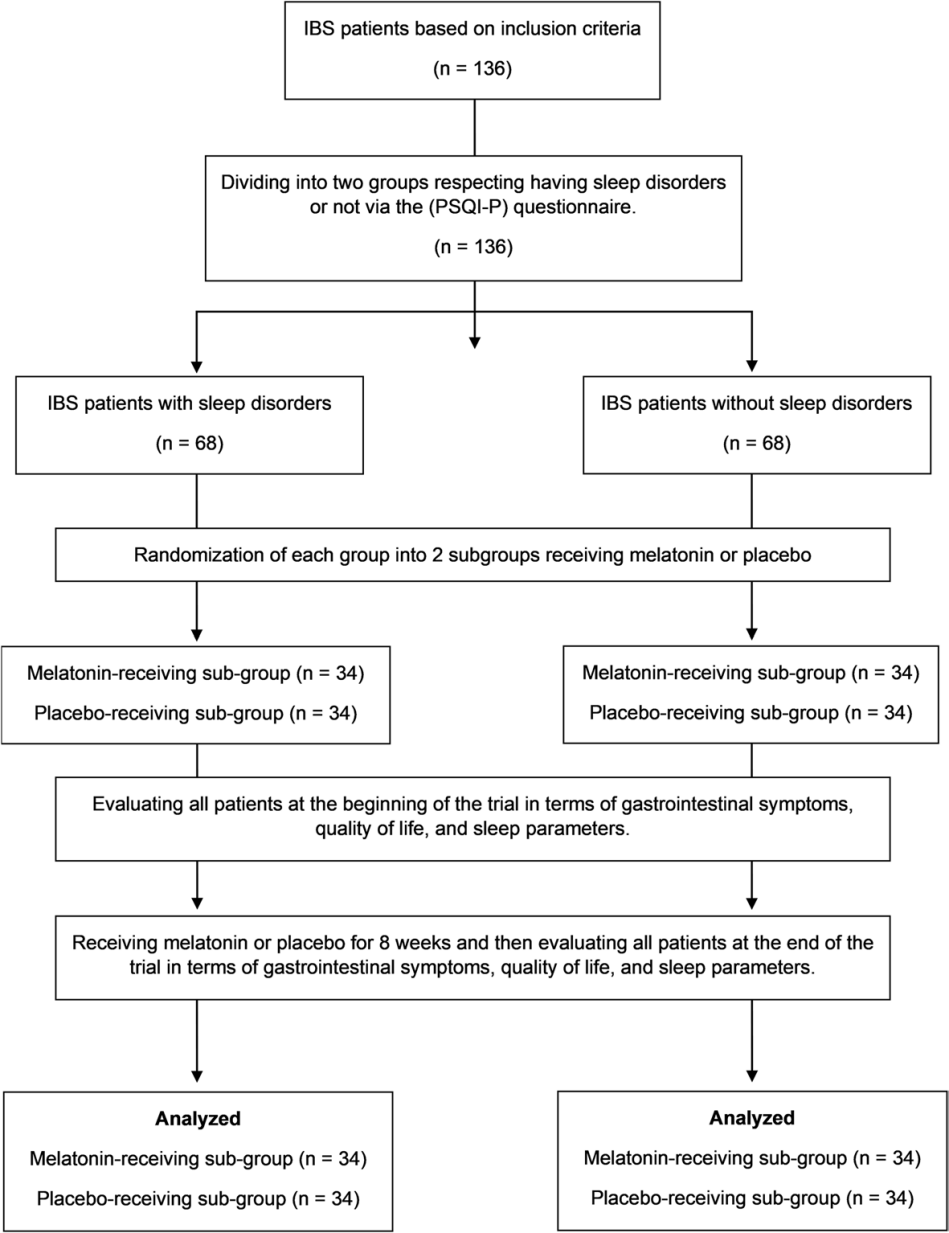
Flow chart of the trial process

This trial was designed and conducted as a randomized, double-blinded, placebo-controlled trial, from February to April 2022, in the gastroenterology clinic of Imam Reza hospital in Tabriz city. In this trial, IBS patients were randomized in a 1:1 ratio to receive 6 mg of melatonin treatment (3 mg fasting and 3 mg at bedtime) or placebo for 8 weeks (2 months). They were evaluated both at the beginning and the end of the trial regarding IBS score, GI symptoms, quality of life, and sleep parameters **(**Fig. 1**)**.

### Sample size

The sample size was calculated via PASS software considering the percentage of patients with mild-to-excellent improvement in IBS symptoms in melatonin recipients (88%) versus placebo recipients (47%) in the study by Lu et al. [5]. Furthermore, in order to calculate the sample size, the statistical power of 80%, confidence interval of 95%, and 20% ratio of dropouts were taken into account. The final sample size was 68 for each group (melatonin or placebo) and 136 in total.

### Inclusion and exclusion criteria

Patients without gender or age limitations with a diagnosis of IBS based on ROME IV criteria (1) without any prior confirmed GI diseases such as inflammatory bowel disease (IBD), (2) without any prior GI surgery, (3) without any history of depression, (4) without using any medications affecting sleep, and (5) without any history of using dietary and herbal supplements, were included. Patients with allergies, side effects, or compromised adherence to the treatment were excluded during the trial.

### Patients

Among the patients who volunteered to participate, 136 were selected, considering the inclusion criteria in a simple block randomization method performed by an independent person who was not involved in the trial. At the beginning of the trial, researchers explained the overview, purpose, trial process, probable side effects, how to withdraw, and any questions of patients. Patients were assured that participation in this trial was voluntary and they could withdraw from the trial at any time without any change in their right to regular treatment. Their participation in this trial and the data they provided would remain confidential; finally, they signed and accepted informed consent papers.

### Blinding

This trial is a double-blind study in which the researchers who completed the questionnaires and patients were unaware of the type of received supplements. When obtaining consent, patients were informed of two types of supplements (melatonin and placebo) but did not know which study groups they would be in. The supplements were provided to patients by another researcher who had no role in completing the questionnaires.

### Interventions

Two types of supplements (melatonin and placebo) were similar, both produced by Zahravi® pharmaceutical company. Supplements were delivered to each patient every two weeks at the gastroenterology clinic, and during delivery, the researcher asked patients whether they had taken previous supplements completely.

### Assessments

First, patients were categorized into two groups, respecting having sleep disorders or not, via the Persian version of the Pittsburgh Sleep Quality Index (PSQI-P) questionnaire [9]. Then, each group was evenly and randomly assigned to get melatonin 6 mg daily (3 mg fasting and 3 mg at bedtime) or placebo. Blocked randomization was used in this process by an independent person not involved in the trial. In each block, patients were matched based on age and gender.

GI symptoms, including the severity of abdominal pain, frequency of abdominal pain in ten days, the severity of abdominal bloating, satisfaction with bowel habits, disease's impact patient’s life, frequency of defecation per week, and stool consistency, were evaluated by researchers through the Irritable Bowel Syndrome Severity Scoring System (IBS-SSS) questionnaire [10], at the beginning and the end of the trial. Researchers evaluated the quality-of-life improvement through the Persian version of the IBS Quality of Life (IBS-QOL-34) questionnaire [11]. Sleep parameters, including subjective sleep quality, sleep latency, sleep duration, sleep efficiency, sleep disturbance, use of sleep medication, and daytime dysfunction, were evaluated by researchers through the PSQI-P questionnaire, at the beginning and the end of the trial.

### Follow-up

At the beginning of the study, patients were informed that if complications or allergies occur during and within one month after the trial, they should stop using the supplements and refer to the gastroenterology clinic as soon as possible.

### Analysis

Collected data were analyzed by SPSS software version 25. Kolmogorov–Smirnov test was used to evaluate the normal distribution of quantitative parameters. Regarding the non-normal distribution of quantitative data, Mann–Whitney U test was used to compare the quantitative data. The Chi-squared or Fisher's exact test was used to compare qualitative parameters. *P*-Value less than 0.05 was assumed as a significant difference.

### Ethics

This trial was approved by the Tabriz University of Medical Sciences ethics committee with the approval number IR.TBZMED.REC.1400.387 on 02/08/2021. Also, this study was registered to the Iranian Registry of Clinical Trials (IRCT) with the approval number IRCT20220104053626N2 on 13/02/2022.

## Results

In this study, 136 patients with IBS were included, of which 68 (50%) had sleep disorders, according to the PSQI score. Each group (with and without sleep disorders) was evenly and randomly divided into two subgroups of 34 patients to receive melatonin or placebo. During the trial, patients tolerated supplements well, and no specific side effects or allergies were reported. In addition, all patients had adherence to the trial. Therefore, we did not exclude any patients, and the drop-out rate was 0%.

Data did not follow a normal distribution. 62 patients were male (45.5%), and 74 were female (54.5%) **(**Table 1). At the beginning of the study, there was no significant difference between melatonin and placebo recipients in both groups with and without sleep disorders regarding demographic data, GI symptoms, and sleep parameters (*P* > 0.05), as shown in Tables 1, 2 and 3.

**Table 1:** Demographic data of the patients

Variable	Group with sleep disorders			Group without sleep disorders		
	**Melatonin**	**Placebo**	***P*-Value**	**Melatonin**	**Placebo**	***P*-Value**
**Number of Patients**	34	34	-	34	34	-
**Age**	39.9 ± 14.6	36.0 ± 11.4	0.296	39.6 ± 13.2	37.2 ± 11.2	0.555
**Gender**						
** Male**	13 (38.2%)	14 (41.2%)	0.804	17 (50%)	18 (52.9%)	0.808
** Female**	21 (61.8%)	20 (58.8%)		17 (50%)	16 (47.1%)	
**Marital Status**						
** Single**	11 (32.4%)	10 (29.4%)	0.793	12 (35.3%)	15 (44.1%)	0.457
** Married**	23 ( 67.6%)	24 (70.6%)		22 (64.7%)	19 (55.9%)	
**Smoking**						
** Yes**	10 (29.4%)	6 (17.6%)	0.253	11 (32.4%)	10 (29.4%)	0.793
** No**	24 (70.6%)	28 (82.4%)		23 (67.6%)	24 (70.6%)

**Table 2:** IBS score and GI symptoms at the beginning and end of the trial

Variable			Group with sleep disorders		Group without sleep disorders	
			**Baseline**	**After Intervention**	**Baseline**	**After Intervention**
**IBS Score**	**Melatonin**	**Mild**	2 (5.8%)	20 (58.8%)	5 (14.7%)	20 (58.8%)
		**Moderate**	16 (47.1%)	9 (26.5%)	13 (38.2%)	9 (26.5%)
		**Severe**	16 (47.1%)	5 (14.7%)	16 (47.1%)	5 (14.7%)
	**Placebo**	**Mild**	5 (14.7%)	10 (29.4%)	2 (5.8%)	8 (23.5%)
		**Moderate**	15 (44.1%)	15 (44.1%)	21 (61.8%)	22 (64.7%)
		**Severe**	14 (41.2%)	9 (26.5%)	11 (32.3%)	4 (11.8%)
	***P*-Value**		0.424	0.022*	0.536	0.025*
**Severity of Abdominal Pain**	**Melatonin**	**No Pain**	0 (0%)	9 (26.5%)	0 (0%)	7 (20.6%)
		**Not Very Severe**	15 (44.1%)	18 (52.9%)	8 (23.5%)	16 (47.1%)
		**Quite Severe**	8 (23.5%)	5 (14.7%)	22 (64.7%)	10 (29.4%)
		**Severe**	8 (23.5%)	2 (5.9%)	4 (11.8%)	1 (2.9%)
		**Very Severe**	3 (8.8%)	0 (0%)	0 (0%)	0 (0%)
	**Placebo**	**No Pain**	0 (0%)	4 (11.8%)	0 (0%)	4 (11.8%)
		**Not Very Severe**	18 (52.9%)	17 (50%)	14 (41.2%)	10 (29.4%)
		**Quite Severe**	6 (17.6%)	5 (14.7%)	14 (41.2%)	17 (50%)
		**Severe**	7 (20.6%)	6 (17.6%)	6 (17.6%)	3 (8.8%)
		**Very Severe**	3 (8.8%)	2 (5.9%)	0 (0%)	0 (0%)
	***P*-Value**		0.575	0.034*	0.384	0.033*
**Frequency of Abdominal Pain in 10 days**	**Melatonin**	**Median**	5.5	3	5	3
		**Q1**	4	0	4	2
		**Q3**	9	6.25	7.25	4.5
	**Placebo**	**Median**	4	4	5	5
		**Q1**	4	3	3	3
		**Q3**	9	8	7	6
	***P*-Value**		0.623	0.019*	0.170	0.010*
**Severity of Abdominal Bloating**	**Melatonin**	**No Bloating**	0 (0%)	8 (23.5%)	0 (0%)	17 (50%)
		**Not Very Severe**	16 (47.1%)	19 (55.9%)	8 (23.5%)	12 (35.3%)
		**Quite Severe**	9 (26.5%)	4 (11.8%)	22 (64.7%)	5 (14.7%)
		**Severe**	7 (20.6%)	3 (8.8%)	4 (11.8%)	0 (0%)
		**Very Severe**	2 (5.9%)	0 (0%)	0 (0%)	0 (0%)
	**Placebo**	**No Bloating**	0 (0%)	2 (5.9%)	1 (2.9%)	7 (20.6%)
		**Not Very Severe**	19 (55.9%)	17 (50%)	14 (41.2%)	14 (41.2%)
		**Quite Severe**	10 (29.4%)	11 (32.4%)	16 (47.1%)	11 (32.3%)
		**Severe**	5 (14.7%)	4 (11.8%)	2 (5.9%)	1 (2.9%)
		**Very Severe**	0 (0%)	0 (0%)	1 (2.9%)	1 (2.9%)
	***P*-Value**		0.292	0.018*	0.261	0.005*
**Satisfaction with Bowel Habits**	**Melatonin**	**Very Happy**	0 (0%)	10 (29.4%)	0 (0%)	5 (14.7%)
		**Quite Happy**	5 (14.7%)	14 (41.2%)	6 (17.6%)	13 (38.2%)
		**Unhappy**	24 (70.6%)	7 (20.6%)	24 (70.6%)	14 (41.2%)
		**Very Unhappy**	5 (14.7%)	3 (8.8%)	4 (11.8%)	2 (5.9%)
	**Placebo**	**Very Happy**	0 (0%)	2 (5.9%)	0 (0%)	0 (0%)
		**Quite Happy**	7 (20.6%)	10 (29.4%)	2 (5.9%)	9 (26.5%)
		**Unhappy**	23 (67.6%)	19 (55.9%)	29 (85.3%)	22 (64.7%)
		**Very Unhappy**	4 (11.8%)	3 (8.8%)	3 (8.8%)	3 (8.8%)
	***P*-Value**		0.512	0.004*	0.436	0.018*
**Disease's Impact on Patient's Life**	**Melatonin**	**Not at All**	0 (0%)	9 (26.5%)	0 (0%)	4 (11.8%)
		**Not Much**	3 (8.8%)	11 (32.3%)	2 (5.8%)	18 (52.9%)
		**Quite a Lot**	18 (52.9%)	10 (29.4%)	22 (64.7%)	10 (29.4%)
		**Completely**	13 (38.2%)	4 (11.8%)	10 (29.4%)	2 (5.8%)
	**Placebo**	**Not at All**	0 (0%)	2 (5.8%)	0 (0%)	0 (0%)
		**Not Much**	2 (5.8%)	7 (20.6%)	5 (14.7%)	10 (29.4%)
		**Quite a Lot**	18 (52.9%)	15 (44.1%)	22 (64.7%)	20 (58.8%)
		**Completely**	14 (41.2%)	10 (29.4%)	7 (20.6%)	4 (11.8%)
	***P*-Value**		0.720	0.004*	0.222	0.003*
**Frequency of Defecations per Week**	**Melatonin**	**Median**	3.5	2	4	2
		**Q1**	2	1	1	1
		**Q3**	4	3	5	4
	**Placebo**	**Median**	2	2	3	2
		**Q1**	1	1	2	2
		**Q3**	4	4	4	3.25
	***P*-Value**		0.165	0.573	0.449	0.361
**Stool Consistency**	**Melatonin**	**Normal**	5 (14.7%)	19 (55.9%)	0 (0%)	21 (61.8%)
		**Not Normal**	29 (85.3%)	15 (44.1%)	34 (100%)	13 (38.2%)
	**Placebo**	**Normal**	3 (8.8%)	5 (14.7%)	2 (5.8%)	5 (14.7%)
		**Not Normal**	31 (91.2%)	29 (85.3%)	32 (94.1%)	29 (85.3%)
	***P*-Value**		0.455	< 0.001*	0.154	0.029*
**Quality of Life Improvement**	**Melatonin**	**Yes**	23 (67.6%)		21 (61.8%)	
		**No**	11 (32.4%)		13 (38.2%)	
	**Placebo**	**Yes**	9 (26.5%)		12 (35.3%)	
		**No**	25 (73.5%)		22 (64.7%)	
	***P*-Value**		0.001*		0.030*

**Table 3:** Sleep parameters at the beginning and end of the trial

Variable			Group with sleep disorders		Group without sleep disorders	
			**Baseline**	**After Intervention**	**Baseline**	**After Intervention**
**Subjective Sleep Quality**	**Melatonin**	**Very good**	0 (0%)	12 (35.3%)	11 (32.4%)	12 (35.3%)
		**Fairly good**	7 (20.6%)	16 (47.1%)	23 (67.6%)	22 (64.7%)
		**Fairly bad**	17 (50%)	6 (17.6%)	0 (0%)	0 (0%)
		**Very bad**	10 (29.4%)	0 (0%)	0 (0%)	0 (0%)
	**Placebo**	**Very good**	0 (0%)	0 (0%)	6 (17.6%)	7 (20.6%)
		**Fairly good**	10 (29.4%)	14 (41.2%)	28 (82.4%)	27 (79.4%)
		**Fairly bad**	14 (41.2%)	10 (29.4%)	0 (0%)	0 (0%)
		**Very bad**	10 (29.4%)	10 (29.4%)	0 (0%)	0 (0%)
	***P*-Value**		0.635	< 0.001*	0.165	0.180
**Sleep Latency**	**Melatonin**	** < 15 min**	0 (0%)	12 (35.3%)	13 (38.2%)	15 (44.1%)
		**16 – 30 min**	8 (23.5%)	17 (50%)	17 (50%)	18 (52.9%)
		**31 – 60 min**	8 (23.5%)	3 (8.8%)	3 (8.8%)	1 (2.9%)
		** > 60 min**	18 (52.9%)	2 (5.9%)	1 (2.9%)	0 (0%)
	**Placebo**	** < 15 min**	0 (0%)	0 (0%)	19 (55.9%)	20 (58.8%)
		**16 – 30 min**	13 (38.2%)	13 (38.2%)	10 (29.4%)	13 (38.2%)
		**31 – 60 min**	5 (14.7%)	5 (14.7%)	5 (14.7%)	1 (2.9%)
		** > 60 min**	16 (47.1%)	16 (47.1%)	0 (0%)	0 (0%)
	***P*-Value**		0.377	< 0.001*	0.270	0.249
**Sleep Duration**	**Melatonin**	** > 7 h**	1 (2.9%)	15 (44.1%)	32 (94.1%)	33 (97.1%)
		**6 – 7 h**	12 (35.3%)	17 (50%)	1 (2.9%)	0 (0%)
		**5 – 6 h**	14 (41.2%)	2 (5.9%)	1 (2.9%)	1 (2.9%)
		** < 5 h**	7 (20.6)	0 (0%)	0 (0%)	0 (0%)
	**Placebo**	** > 7 h**	0 (0%)	0 (0%)	33 (97.1%)	33 (97.1%)
		**6 – 7 h**	16 (47.1%)	16 (47.1%)	0 (0%)	0 (0%)
		**5 – 6 h**	11 (32.4%)	11 (32.4%)	1 (2.9%)	1 (2.9%)
		** < 5 h**	7 (20.6)	7 (20.6)	0 (0%)	0 (0%)
	***P*-Value**		0.669	< 0.001*	0.569	1.000
**Sleep Efficiency**	**Melatonin**	** > 85%**	10 (29.4%)	26 (76.5%)	33 (97.1%)	33 (97.1%)
		**75 – 84%**	11 (32.4%)	5 (14.7%)	1 (2.9%)	1 (2.9%)
		**65 – 74%**	10 (29.4%)	3 (8.8%)	0 (0%)	0 (0%)
		** < 65%**	3 (8.8%)	0 (0%)	0 (0%)	0 (0%)
	**Placebo**	** > 85%**	9 (26.5%)	9 (26.5%)	34 (100%)	34 (100%)
		**75 – 84%**	10 (29.4%)	10 (29.4%)	0 (0%)	0 (0%)
		**65 – 74%**	13 (38.2%)	13 (38.2%)	0 (0%)	0 (0%)
		** < 65%**	2 (5.9%)	2 (5.9%)	0 (0%)	0 (0%)
	***P*-Value**		0.748	< 0.001*	0.317	0.317
**Sleep Disturbance**	**Melatonin**	**Normal**	1 (2.9%)	5 (14.7%)	30 (88.2%)	30 (88.2%)
		**Mild**	30 (88.2%)	28 (82.4%)	4 (11.8%)	4 (11.8%)
		**Moderate**	3 (8.8%)	1 (2.9%)	0 (0%)	0 (0%)
		**Severe**	0 (0%)	0 (0%)	0 (0%)	0 (0%)
	**Placebo**	**Normal**	3 (8.8%)	3 (8.8%)	32 (94.1%)	32 (94.1%)
		**Mild**	28 (82.4%)	28 (82.4%)	2 (5.9%)	2 (5.9%)
		**Moderate**	3 (8.8%)	3 (8.8%)	0 (0%)	0 (0%)
		**Severe**	0 (0%)	0 (0%)	0 (0%)	0 (0%)
	***P*-Value**		0.537	0.251	0.396	0.396
**Use of Sleep Medication**	**Melatonin**	**Not during past month**	34 (0%)	34 (0%)	34 (100%)	34 (100%)
		**Less than once a week**	0 (0%)	0 (0%)	0 (0%)	0 (0%)
		**Once or twice a week**	0 (0%)	0 (0%)	0 (0%)	0 (0%)
		**Three or more times a week**	0 (0%)	0 (0%)	0 (0%)	0 (0%)
	**Placebo**	**Not during past month**	34 (100%)	34 (100%)	34 (100%)	34 (100%)
		**Less than once a week**	0 (0%)	0 (0%)	0 (0%)	0 (0%)
		**Once or twice a week**	0 (0%)	0 (0%)	0 (0%)	0 (0%)
		**Three or more times a week**	0 (0%)	0 (0%)	0 (0%)	0 (0%)
	***P*-Value**		1.000	1.000	1.000	1.000
**Daytime Dysfunction**	**Melatonin**	**Normal**	0 (0%)	6 (17.6%)	12 (35.3%)	14 (41.2%)
		**Slight Dysfunction**	13 (38.2%)	21 (61.8%)	22 (64.7%)	20 (58.8%)
		**Moderate Dysfunction**	17 (50%)	6 (17.6%)	0 (0%)	0 (0%)
		**Severe Dysfunction**	4 (11.8%)	1 (2.9%)	0 (0%)	0 (0%)
	**Placebo**	**Normal**	0 (0%)	0 (0%)	17 (50%)	18 (52.9%)
		**Slight Dysfunction**	11 (32.4%)	13 (38.2%)	16 (47.1%)	16 (47.1%)
		**Moderate Dysfunction**	18 (52.9%)	16 (47.1%)	1 (2.9%)	0 (0%)
		**Severe Dysfunction**	5 (14.7%)	5 (14.7%)	0 (0%)	0 (0%)
	***P*-Value**		0.583	< 0.001*	0.294	0.335

In the group with sleep disorders, 32 melatonin (94.1%) and 29 placebo recipients (85.3%) had moderate and severe IBS, reduced to 14 (41.2%) and 24 patients (70.6%) at the end of the trial, respectively (*P*-Value = 0.022); 19 melatonin (55.9%) and 16 placebo recipients (47.1%) complained of quite severe to very severe abdominal pain, reduced to 7 (20.6%) and 13 patients (38.2%), respectively (*P*-Value = 0.034); 18 melatonin (52.9%) and 15 placebo recipients (44.1%) complained of quite severe to very severe abdominal bloating, reached to 7 (20.6%) and 15 patients (44.1%), respectively (*P*-Value = 0.018); 29 melatonin (85.3%) and 27 placebo recipients (79.4%) were unhappy with their bowel habits, reduced to 10 (29.4%) and 22 patients (64.7%), respectively (*P*-Value = 0.004); 31 melatonin (91.2%) and 32 placebo recipients (94.1%) stated that the disease had affected their lives, which was reduced to 14 (41.2%) and 25 patients (73.5%), respectively (*P*-Value = 0.004); 5 melatonin (14.7%) and 3 placebo recipients (8.8%) had normal stool consistency, which was reported normal in 19 (55.9%) and 5 patients (14.7%) at the end of the trial, respectively (*P*-Value < 0.001). In addition, the number of days with abdominal pain in ten days was significantly reduced in melatonin recipients compared to placebo (*P*-Value = 0.019); however, there were no significant changes in the frequency of defecation per week in these patients (*P*-Value = 0.573). Finally, 23 melatonin recipients (67.6%) expressed quality-of-life improvement, while 9 placebo recipients (26.5%) expressed (*P*-Value = 0.001) (Table 2).

In the group without sleep disorders, 29 melatonin (85.3%) and 32 placebo recipients (94.1%) had moderate and severe IBS, reduced to 14 (41.2%) and 26 patients (76.4%) at the end of the trial, respectively (*P*-Value = 0.025); 26 melatonin (76.4%) and 20 placebo recipients (58.8%) complained of quite severe to very severe abdominal pain, reached to 11 (32.3%) and 20 patients (58.8%), respectively (*P*-Value = 0.033); 26 melatonin (76,4%) and 19 placebo recipients (55.9%) complained of quite severe to very severe abdominal bloating, reduced to 5 (14.7%) and 13 patients (38.2%), respectively (*P*-Value = 0.005); 28 melatonin (82.3%) and 32 placebo recipients (94.1%) were unhappy with their bowel habits, reached to 16 (47.1%) and 25 patients (73.5%), respectively (*P*-Value = 0.018); 32 melatonin (94.1%) and 29 placebo recipients (85.3%) stated that the disease had affected their lives, reduced to 12 (35.3%) and 24 patients (70.5%), respectively (*P*-Value = 0.003); 0 melatonin recipients (0%) and 2 placebo recipients (5.8%) had normal stool consistency, which was reported normal in 21 (61.8%) and 5 patients (14.7%) at the end of the trial, respectively (*P*-Value = 0.029). In addition, the number of days with abdominal pain in ten days was significantly reduced in melatonin recipients compared to placebo (*P*-Value = 0.010); however, there were no significant changes in the frequency of defecation per week in these patients (*P*-Value = 0.361). Finally, 21 melatonin recipients (61.8%) expressed quality-of-life improvement, while 12 placebo recipients (35.3%) expressed (*P*-Value = 0.030) (Table 2).

In the group with sleep disorders, 27 melatonin (79.4%) and 24 placebo recipients (70.5%) had fairly and very bad subjective sleep quality, reduced to 6 (17.6%) and 20 patients (58.8%) at the end of the trial, respectively (*P*-Value < 0.001); 26 melatonin (76.4%) and 21 placebo recipients (61.7%) had a sleep latency of more than 30 min, reached to 5 (14.7%) and 21 patients (61.7%), respectively (*P*-Value < 0.001); 21 melatonin (61.7%) and 18 placebo recipients (52.9%) had sleep duration less than 6 h, reached to 2 (5.8%) and 18 patients (52.9%), respectively (*P*-Value < 0.001); 13 melatonin (38.2%) and 15 placebo recipients (44.1%) had sleep efficiency less than 75%, reached to 3 (8.8%) and 15 patients (44.1%), respectively (*P*-Value < 0.001); 33 melatonin (97.1%) and 31 placebo recipients (91.2%) had mild to severe sleep disturbance, reached to 29 (85.3%) and 31 patients (91.2%), respectively (*P*-Value = 0.251); 21 melatonin (61.7%) and 23 placebo recipients (67.6%) had moderate to severe daytime dysfunction, reached to 7 (20.6%) and 21 patients (61.8%), respectively (*P*-Value < 0.001) (Table 3).

In the group without sleep disorders, 11 melatonin (32.4%) and 6 placebo recipients (17.6%) had very good subjective sleep quality, increased to 12 (35.3%) and 7 patients (20.6%) at the end of the trial, respectively (*P*-Value = 0.180); 30 melatonin (88.2%) and 29 placebo recipients (85.2%) had a sleep latency of less than 30 min, increased to 33 (97.1%) and 33 patients (97.1%), respectively (*P*-Value = 0.249); 32 melatonin (94.1%) and 33 placebo recipients (97.1%) had sleep duration more than 7 h, reached to 33 (97.1%) and 33 patients (97.1%), respectively (*P*-Value = 1.000); 33 melatonin (97.1%) and 34 placebo recipients (100%) had sleep efficiency more than 85%, which remained unchanged (*P*-Value = 0.317); 30 melatonin (88.2%) and 32 placebo recipients (94.1%) did not have sleep disturbance which remained unchanged (*P*-Value = 0.396); 22 melatonin (64.7%) and 17 placebo recipients (50%) had daytime dysfunction, reached to 20 (58.8%) and 16 patients (47.1%), respectively (*P*-Value = 0.335) (Table 3).

## Discussion

IBS is a concerning and common disease worldwide that significantly burdens the health system as a chronic situation. Clinical manifestations vary among individuals, and current treatments do not meet patients’ demands [12]. In this trial, we studied the effect of melatonin 6 mg daily (3 mg fasting and 3 mg at bedtime) for 2 months (8 weeks) compared to placebo, on the improvement of IBS score, GI symptoms, quality of life, and sleep parameters in both groups of IBS patients with and without sleep disorders.

Melatonin is a hormone secreted by the pineal gland with a photoperiodic pattern that regulates the circadian rhythm. Furthermore, melatonin is also found in the GI tract, 400 times more than the secreted amount of the pineal gland, which is mostly synthesized in enterochromaffin cells [4, 12]. Mozaffari et al. [4] conducted a systematic review on the effects of melatonin in the GI tract and IBS; and concluded that melatonin has anxiolytic, anti-inflammatory, anti-oxidative, and motility regulatory effects. In addition, they concluded that melatonin concentrations are disturbed in IBS patients so melatonin deficiency may be an influential factor in the pathogenesis of IBS. They suggested that exogenous melatonin may benefit these patients by reducing abdominal pain and improving overall IBS symptom scores due to its potential to regulate GI motility.

Furthermore, sleep disorders are discussed as another possible influencing factor in the pathogenesis of IBS. Wang et al. [13] performed a systematic review and meta-analysis in this field; and concluded that sleep disorders are more common among IBS patients than healthy controls, so circadian rhythm disorders and related factors may be influential in the pathogenesis of IBS. As mentioned, circadian rhythm is mainly regulated by the melatonin hormone. Since melatonin has been successfully used in treating sleep disorders such as insomnia [14], it can also be used in IBS patients.

IBS affects not only patients' physical health but also various aspects of daily life, including psychological health, work productivity, and social functioning. It is associated with life-disrupting symptoms such as abdominal pain, changes in bowel habits, bloating, fibromyalgia, chronic pelvic pain, and chronic fatigue syndrome. Since IBS is a chronic disease with a relatively long diagnostic and therapeutic course, patients are prone to poor quality of life [3, 12, 15].

We included 136 IBS patients, which was more than similar trials [5–8]. We did not apply any gender or age limitations to the inclusion criteria; however, Lu et al. [5] studied only female patients; and Chojnacki et al. [8] studied only postmenopausal female patients. In this trial, patients were diagnosed based on ROME IV criteria which was different from other trials, three of them [5–7] used ROME II criteria, and Chojnacki et al. [8] used ROME III criteria. The ROME criteria were first developed in the 1990s, then underwent three revisions, with the latest being published in 2016 as ROME IV [16]. Black et al. [16] showed that Rome IV criteria are significantly more specific than Rome III criteria in diagnosing IBS. The dose of melatonin received by the patients was 6 mg daily (3 mg fasting and 3 mg at bedtime), which was higher than most similar studies [5–7]. They investigated the effect of melatonin 3 mg at bedtime; however, Chojnacki et al. [8] studied the effect of 3 mg fasting, and 5 mg at bedtime. The duration of the present trial was 8 weeks, which was similar to the trials by Lu et al. [5] and Saha et al. [7]; more than the trial by Song et al. [6] which was 2 weeks; and less than the trial by Chojnacki et al. [8] which was 6 months.

Chen et al. [17] performed a meta-analysis study to investigate the efficacy of melatonin supplements in improving IBS severity in IBS patients, and they included four trials with 115 IBS patients [5–8]. They concluded that melatonin supplement was associated with a significant improvement in overall IBS severity, pain severity, and quality of life; however, there were no significant improvements in abdominal bloating and sleep quality.

The results of our trial showed that in both groups of patients with and without sleep disorders, receiving melatonin leads to a significant improvement in IBS score and GI symptoms, including the severity and the frequency of abdominal pain, the severity of abdominal bloating, satisfaction with bowel habits, disease's impact on patient's life, and stool consistency; however, there was no significant improvement in the frequency of defecations per week. In patients with sleep disorders, significant improvement in sleep parameters, including subjective sleep quality, sleep latency, sleep duration, sleep efficiency, and daytime dysfunction, was observed, while in patients without sleep disorders, there was no significant improvement in sleep parameters. In addition, quality-of-life improvement was observed in a significant number of melatonin recipients compared to placebo in both groups of patients.

The differences between the results of our trial and other trials may be due to the difference in the trial duration, melatonin dosage, and scales used for evaluating the IBS symptoms, sleep parameters, and quality of life. In addition, they evaluated some parameters that we did not, such as psychological profile, colonic transit times, polysomnographic parameters, extracolonic parameters, and rectal sensory thresholds. In the following, we will discuss the studied parameters and results of each trial.

Lu et al. [5] studied parameters such as overall IBS Score, IBS symptoms (abdominal pain, abdominal bloating, abnormal sensation of defecation, stool consistency, and frequency of defecation), sleep parameters, anxiety, depression, well-being, and colonic transit times, in female patients. They concluded that using melatonin 3 mg at bedtime for 8 weeks compared to placebo led to a significant reduction in the overall IBS Score and IBS symptoms, including abdominal pain, abdominal bloating, and abnormal sensation of defecation. However, melatonin did not significantly affect stool consistency, frequency of defecation, sleep parameters, psychological profiles, and colonic transit times.

Song et al. [6] studied parameters such as overall IBS Score, IBS symptoms (abdominal pain, abdominal bloating, abnormal sensation of defecation, stool consistency, and frequency of defecation), quality of life, polysomnographic parameters, sleep parameters (sleep quality, sleep latency, sleep duration, sleep efficiency, sleep disturbance, use of sleep medications, and daytime dysfunction), anxiety, depression, and rectal sensory thresholds. They concluded that using melatonin 3 mg at bedtime for 2 weeks compared to placebo led to a significant improvement in abdominal pain, and rectal pain sensitivity. However, melatonin did not significantly affect overall IBS Score, abdominal bloating, abnormal sensation of defecation, stool consistency, frequency of defecation, quality of life, polysomnographic parameters, sleep parameters, and psychological profiles.

Saha et al. [7] studied parameters such as overall IBS Score, IBS symptoms (abdominal pain and frequency, abdominal bloating, dissatisfaction with bowel habits, life interference), overall extracolonic score (nausea, vomiting, headaches, thigh pain, body aches, backaches, early satiety, excess wind, heartburn, urinary symptoms, and lethargy), and quality of life (psychic well-being, physical well-being, mood, social relationship, locus of control, and coping with work). They concluded that using melatonin 3 mg at bedtime for 8 weeks compared to placebo significantly improved overall IBS score, IBS symptoms, extracolonic IBS score, and overall quality-of-life score.

Chojnacki et al. [8] studied IBS symptoms such as visceral pain, abdominal bloating, and constipation or diarrhoea in postmenopausal women with IBS in two groups of constipation-predominant IBS (IBS-C) and diarrhea-predominant IBS (IBS-D). They concluded that using melatonin (3 mg fasting and 5 mg at bedtime) for 6 months improves the IBS symptoms significantly in all patients and patients with IBS-C; however, this improvement was not significant in patients with IBS-D.

### Limitations

The most important limitation of our trial was not classifying the sub-types of IBS (IBS-C or IBS-D), which probably affected the results of the "Frequency of defecations per week" parameter. Adherence to the trial was evaluated based on the patient's statements so that all patients stated that they adhered to the trial, and no patients were excluded from the trial for this reason. Another limitation was using questionnaires to evaluate the parameters because some patients may not be accurate enough or even exaggerate when answering the questions; so, using other methods, such as polysomnography, could give more accurate data instead of a questionnaire to determine sleep disorders. For a definite conclusion as well as the optimal drug dose, patients who benefit the most from this treatment, and long-term effects of melatonin, it is recommended conduct more trials with a larger sample size in which patients are classified into IBS subtypes, in a more extended period of trial using objective methods than questionaries.

## Conclusion

Melatonin can be considered an effective treatment for improving IBS score, GI symptoms, and quality of life in IBS patients with and without sleep disorders. It is also effective to improve sleep parameters in IBS patients with sleep disorders.

## Data Availability

The datasets used and/or analyzed during the current study are available from the corresponding author on reasonable request.

## Abbreviations

GI: Gastrointestinal
IBD: Inflammatory Bowel Disease
IBS: Irritable Bowel Syndrome
IBS-C: Constipation-Predominant IBS
IBS-D: Diarrhea-Predominant IBS
IBS-SSS: Irritable Bowel Syndrome Severity Scoring System
IBS-QOL: Irritable Bowel Syndrome Quality Of Life
IRCT: Iranian Registry Of Clinical Trials
PSQI-P: Pittsburgh Sleep Quality Index-Persian
